# Maintenance and recall of memory T cell populations against tuberculosis: Implications for vaccine design

**DOI:** 10.3389/fimmu.2023.1100741

**Published:** 2023-03-31

**Authors:** Xin Liu, Haoran Li, Shanshan Li, Jinfeng Yuan, Yu Pang

**Affiliations:** Department of Bacteriology and Immunology, Beijing Chest Hospital, Capital Medical University/Beijing Tuberculosis and Thoracic Tumor Research Institute, Beijing, China

**Keywords:** *Mycobacterium tuberculosis*, memory T cells, vaccine, host immune responses, tuberculosis

## Abstract

Despite the widespread use of standardised drug regimens, advanced diagnostics, and *Mycobacterium bovis* Bacille-Calmette-Guérin (BCG) vaccines, the global tuberculosis (TB) epidemic remains uncontrollable. To address this challenge, improved vaccines are urgently required that can elicit persistent immunologic memory, the hallmark of successful vaccines. Nonetheless, the processes underlying the induction and maintenance of immunologic memory are not entirely understood. Clarifying how memory T cells (Tm cells) are created and survive long term may be a crucial step towards the development of effective T cell–targeted vaccines. Here, we review research findings on the memory T cell response, which involves mobilization of several distinct Tm cell subsets that are required for efficient host suppression of *M. tuberculosis* (Mtb) activity. We also summaries current knowledge related to the T cell response-based host barrier against Mtb infection and discuss advantages and disadvantages of novel TB vaccine candidates.

## Introduction

Tuberculosis (TB), which is caused by infection with *Mycobacterium tuberculosis* (Mtb), is a global public health emergency. An estimated one-third of the world’s population is afflicted with some form of Mtb infection, while each year approximately 10 million active patients and over 1.5 million die of the disease (1). Vaccines are recognised as one of the most successful tools for combating bacterial and viral infectious diseases. In fact, the highly effective smallpox vaccine has completely eradicated one of the most devastating and deadly infectious diseases to afflict mankind (2). Additionally, the Bacillus Calmette-Guérin (BCG) vaccine, the only approved anti-TB vaccine, has saved millions of lives by preventing childhood TB infection, but has limited effectiveness for TB prevention in adults. Therefore, there is an urgent need to develop new and improved TB vaccines that can provide more robust and durable protection against Mtb infection.

The host immune response is critically important for controlling infections caused by intracellular pathogens, including viral, bacterial, and parasitic organisms. This infection-controlling response depends on memory T cell (Tm) longevity, which is clearly responsible for the long-term success of smallpox and poliovirus vaccines (3). Tm cells are generated in the host response to initial infection that triggers naive T cells to clonally expand when stimulated by foreign antigen at the infection site. Concurrently, clonally expanding T cells differentiate into effector T cells that participate in infection clearance. After the infection is cleared, lack of antigenic stimulation causes the T effector population at the infection site to clonally contract. Next, some cells in the remaining population survive and develop into Tm cells, which are critically important for long-term immunity, since they can initiate a rapid recall response during future encounters with the same pathogen. In contrast to naive T cells, Tm cells possess a lower activation threshold that enables them to more rapidly differentiate into effector cells. Furthermore, Tm cells possess greater ability to migrate to lymph nodes and remain there, which reduces Tm response time and prolongs Tm-mediated protection against infection. Importantly, the initial effector T cell clonal burst size may determine the size of the Tm cell pool. Thus, an effective vaccine should induce an effector T cell population that is as large as possible in order to maximise the size of the effector-derived Tm cell population, since it is well known that T cell response strength correlates strongly with infection control effectiveness. Hence, one promising approach for achieving vaccination-induced TB control entails the generation of greater numbers of longer-lived Tm cells. However, Mtb has developed various strategies to evade, delay, and inhibit T cell responses that include induction of increased regulatory T cell (Treg cell) activity and T cell exhaustion. These and additional challenges (e.g., target antigen selection and prevention of chronic antigen stimulation) must be overcome before safe vaccines can be developed that generate effective long-lived Tm cell responses. In this review, we summarise current knowledge regarding the biology of Tm cells (including their origin and Tm cell subsets), Mtb mechanisms that block T cell immune function, and the key role played by Tm in combating Mtb infection. Within this context, advantages, disadvantages, and challenges related to new candidate anti-Mtb vaccines are discussed.

## Host development of effector and memory T cells

**Figure 1:**
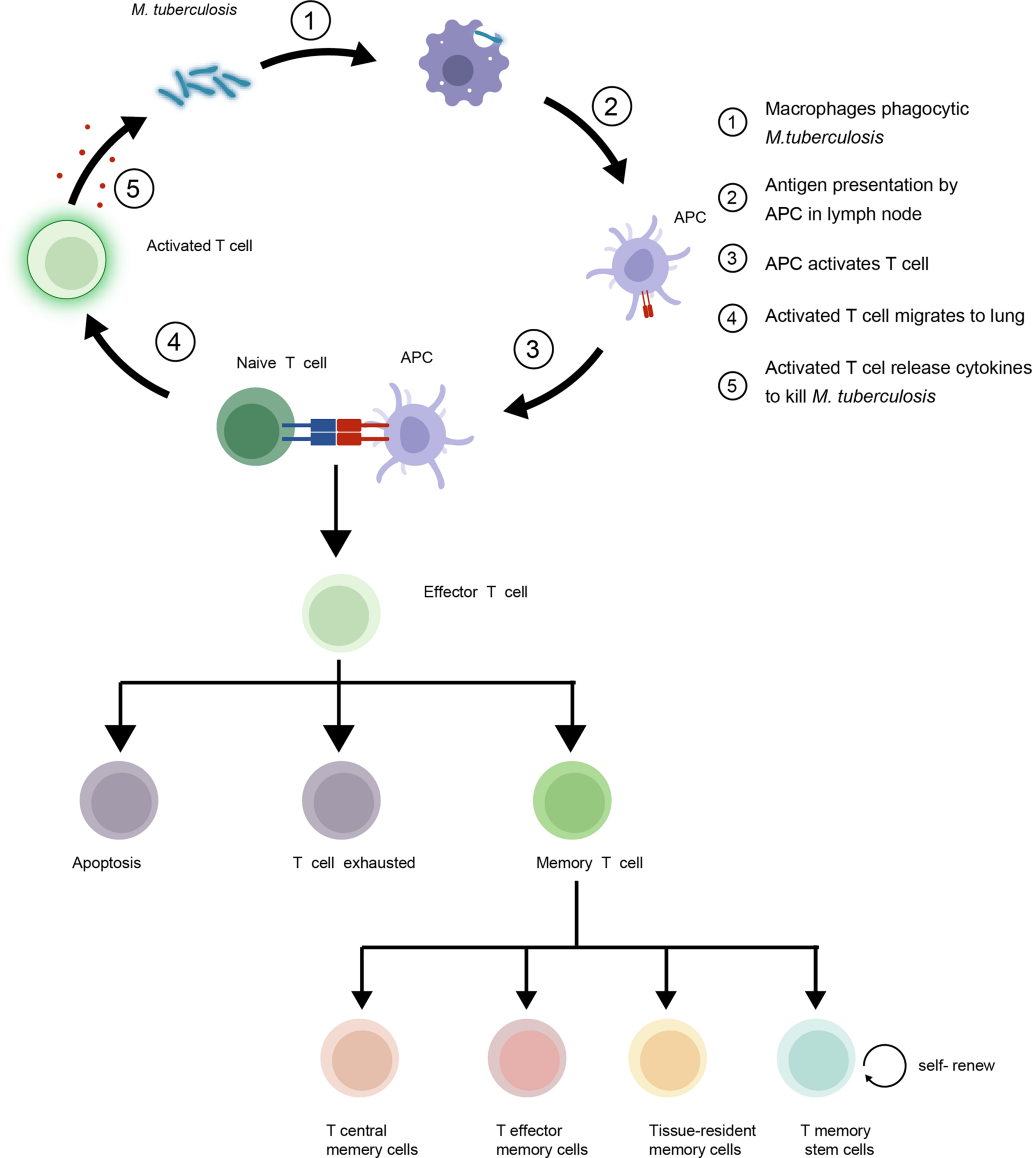
*Mycobacterium tuberculosis* (Mtb) interacts with host T cells and subsets of memory T cells in the immune response to tuberculosis (TB). Upon infection, naive T cells recognize Mtb antigens and differentiate into effector T cells to control growth of the bacteria. Some effector T cells become memory T cells, providing long-term protection against re-infection. The four main subsets of memory T cells are central memory T cells (TCM), effector memory T cells (TEM), Tissue-resident memory T cells (TRM) and T memory stem cells (TSCM).

T cell immune responses play a critical role in mediating adaptive immunity to control and eradicate foreign pathogens. Three main events occur after an infection initially arouses the T cell immune response of the host. First, dendritic cells (DCs) and macrophages act as classical antigen-presenting cells (APCs). APCs are required to convert complex protein antigens into antigenic peptides that can bind to major histocompatibility complex (MHC) I or II proteins to form intracellular MHC I or II protein-antigen complexes. These MHC-antigen complexes then migrate to APC surfaces, where they present antigens to T cells by specifically binding to T cell receptors (TCRs) on T cell surfaces. Interactions between MHC-antigen complexes and TCRs then trigger T cell signaling that activates naive T cells to proliferate and differentiate into effector T cells with pathogen-fighting functions. Next, effector T cells combat intracellular pathogens *via* two mechanisms: through direct killing of infected cells and through production of cytokines that activate other types of effector cells to kill and remove infected cells and antigenic stimuli. Thereafter, in the absence of antigen stimulation, most effector T cells undergo apoptosis that leads to their removal from host tissues as a mechanism for preventing excessive inflammatory responses. This clonal constriction of the effector T cell repertoire appears to result from the loss of pro-survival cytokines (e.g., IL-2) that were produced in response to antigenic stimulation and low cytokine receptor levels on unstimulated effector T cells. Nevertheless, some effector T cells survive and differentiate into Tm cells (4) (**Figure 1**). Hence, the effective generation of immunological memory depends on two extremely important criteria: the number of naive T cells that differentiate into effector cells and the number of effector cells that differentiate into Tm cells.

Throughout life, host T cells undergo significant age-related changes in number and function that vary according to life stage (5). During childhood, naive T cells encounter large numbers of unique antigens that trigger massive antigen-specific T cell expansion followed by sustained production and rapid accumulation of Tm cells. Importantly, antigen diversity continually influences the shaping of host immune responses through adulthood, when Tm cell accumulation slows and reaches a plateau. Thereafter, the memory T cell population enters a state of prolonged homeostasis that initially confers reduced pathogen susceptibility to adults as compared to that of children. However, during later life stages, Tm cell homeostasis slows, leading to changes in proportions of T cell subsets and functionality as Tm cells enter senescence, a state of increased inflammatory responsiveness and decreased cellular function. Notably, activated T cell activities (TCR signaling strength and duration, metabolic activity, cytokine release, and transcription factor expression) all impact T cell development throughout life (6).

## Description of memory T cell subsets and implications for TB vaccine design

Based on cell surface markers and key functional traits, Tm cells can be classified into distinct subsets, including central memory (TCM), effector memory (TEM), and tissue-resident memory (TRM) T cells (**Table 1**). TCMs are CD45R0+ memory cells that constitutively express CCR7 and CD62L, whereas TEMs lack constitutive CCR7 expression, vary in CD62L expression (7), and migrate to infected peripheral tissues to exert immediate protective effector functions. Importantly, reactive memory is mediated by TCMs, which are found in T cell areas of secondary lymphoid organs and have little or no effector function. However, these cells are able to proliferate and rapidly differentiate into effector cells when exposed to foreign antigens (8). As a result, TCMs can provide long-term protection against pathogens, whereas only short-term protection is provided by TEMs. In fact, in patients with active pulmonary TB, a large proportion of TCMs IFN-γ+& TNFα+ are able to mount a protective immune response against Mtb as evidence for their key protective role in eliminating infection in these patients (9). With antigenic stimulations, the frequencies of TCMs are dramatically higher, whereas TEMs are lower in healthy subjects compared to TB disease (10). In active TB, TEMs hold a predominant position, while TCMs predominate in LTBI (11). The relative frequencies of memory cells are thought to highly correlated with changes in antigen load (12).

**Table 1:** Subsets of memory T cells.

Cell type	Surface markers	Location	Duration of protection
T_SCM_	CD45RO-, CD45RA+, CCR7+, CD62L+, CD127+, CD95+, CD122+, CXCR3+	Circulation	Long-term protection
T_CM_	CD45RO+, CD45RA-, CCR7+, CD62L+, CD95+	Circulation lymph nodes	Long-term protection
T_EM_	CD45RO+, CD45RA-, CCR7-, CD62L-, CD95+	Circulation	Short-term protection
T_RM_	PD-1, CD44, CD101, CD69	Tissue resident	Long-term protection

TRMs comprise a non-circulating Tm cell subpopulation. that resides in peripheral tissues, such as the lungs, where they provide local protection against re-infection. TRMs can be distinguished from circulating Tm cells by their high-level cell surface expression of CD69 and CD103 molecules (27), while their functions and transcriptional expression profiles resemble corresponding features of TCM and TEM cells (28). Importantly, TRMs that produce TNF-α, IL-2, and IL-17 undergo rapid proliferation in Mtb-infected lung tissues, meanwhile addition of either exogenous IL-17 or IL-2 to Mtb-infection model has been shown to strengthen the anti-Mtb immune response. Taken together, these observations highlight the essential role played by TRMs in Mtb infection control (29). Moreover, Mtb invasion of host tissues triggers a rapid TRM cell response to infection that activates TRM effector functions (30), whereby Bull et al. found that BCG vaccination led to prolonged CD4+ TRM cell intravascular residence in pulmonary tissues, as evidenced by detection of stained CD4+ TRM cells in lung tissues for at least 12 months post-vaccination.

Despite the fact that TRM cells remove invading pathogens, production of pro-inflammatory substances by these cells may damage normal cells, resulting in asthma and/or fibrosis (31, 32). Likewise, lung TRM cells derived from invading recipient T cells may mediate allograft immunopathology and exacerbate lung damage (33). Hence, even though vaccines can be designed to increase host production of high numbers of TRMs that may act locally to prevent lung infection, special care must be taken to ensure that these vaccines do not induce excessive TRM production.

T memory stem cells (TSCM), a novel memory T cell subset found in humans and mice, initially attracted attention of researchers who observed that these cells could maintain graft-versus-host disease after repeated transplantation in allogeneic hosts (34, 35). This ‘stem cell-like’ property was subsequently found to result from their high proliferative capacity and ability to continually maintain their population size through self-renewal. In addition, TSCMs were observed to differentiate into other T cell subsets, including TCMs and TEMs. Importantly, antigen-specific CD4+ TSCMs induced by Mtb infection have been shown to exert effector functions, including secretion of various Th1 cytokines and expression of chemokine receptors, as have been reported for memory Th1/17 cells (36). Thus, TSCMs hold promise as preventative and therapeutic tools for use in curing disease and providing long-term protection against a variety of illnesses, including cancer and infections. Nonetheless, before the full potential of TSCMs can be harnessed for clinical applications, numerous issues must be resolved.

The most striking and unique feature of TSCMs is their ability to differentiate into TCMs or TEMs that can continuously undergo self-renewal. In turn, the TCM : TEM ratio is thought to influence vaccination effectiveness, since constituent Tm cell subsets perform functions that include a TCM long-term protective function, as was confirmed experimentally through adoptive transfer of TCMs and TEMs (37). Although researchers hope to achieve long-term protection from TB by boosting TCM numbers using BCG and candidate vaccines, TCM numbers may decrease over time, due to chronic environmental Mtb exposure, which reduces TB vaccine efficacy. Thus, Mtb exposure poses a threat to patients living in regions of high TB prevalence (38). Moreover, chronic exposure to environmental Mtb eventually induces antigen-specific T cell anergy and exhaustion (34). Hence, maximizing vaccine-induced CD4+ TSCMs production should be adopted by vaccine designers to strategically enhance long-lasting T cell memory against Mtb antigens.

To address a different challenge, current knowledge related to Tm populations may help explain the variable efficacy of BCG vaccines in children versus adults. First, childhood BCG vaccination induces the generation of a large TEM population that appears to confer protection against TB for 10–15 years (39), as supported by experiments demonstrating that BCG vaccination of mice boosted TEM numbers in lung tissues (40). Second, childhood is a key period of Tm cell generation, since BCG vaccination of infants shortly after birth can induce production of large numbers of antigen-specific Tm cells with protective activities against Mtb infection. However, throughout life environmental Mtb exposures occur that continuously deplete the Tm pool to ultimately weaken BCG vaccine-induced protection from active TB infection. Third, BCG, a live vaccine, contains organisms that likely share antigens with environmental Mtb, such that sustained sensitization to environmental Mtb may hinder development of BCG vaccination-induced TB protection in adults. To address this issue, researchers have developed methods and drugs to promote TCM production to thereby enhance BCG vaccine efficacy. One such method entails supplementation of vaccines with proteins associated with ESX secretion systems (ESX 1-5), which are specialized type VII secretion systems (T7SSs) present in mycobacteria and other organisms. Importantly, guinea pigs and mice vaccinated with BCG supplemented with Mtb ESX-5 acquire increased resistance to extremely pathogenic Mtb strains, which in mice was found to correlate with enhanced TCM inflow into infected tissues (41). In addition, clofazimine (CFZ), a lipophilic riminophenazine antibiotic that has been recommended by the World Health Organisation (WHO) for use as a second-line anti-tuberculosis drug (42), exhibits immunomodulatory properties when administered to BCG-vaccinated mice. Specifically, CFZ has been shown to expand the TCM population to enhance the TCM response, while concurrently blocking TEM cell surface Kv1.3 potassium channels to reduce TEM cell number. Ultimately, these effects increase the TCM : TEM ratio to improve vaccine efficacy (36, 43).

## The T cell-mediated response to Mtb infection

Mtb infection can effectively induce T cell immunity as an antibacterial response that is mediated by both CD4+ and CD8+T cells (44). CD8+T cells can kill Mtb-infected cells either by secreting perforin, granzymes, and granulysin or by engaging in apoptosis-inducing Fas-Fas ligand interactions. However, these activities provide limited protection against infection. By contrast, CD4+ T cells exert their primary protective effect by secreting a range of cytokines, such as IFN-γ and TNF-α, which draw other immune cells to the infection site to promote differentiation of several CD4+ T cell subsets into effector cells that can eliminate Mtb. In fact, results of numerous studies emphasise the critical role played by CD4+ T cells in host anti-Mtb defence. For example, studies have demonstrated that downregulation of CD4+ T cells, a significant feature of HIV infection, can lead to dramatically increased rates of latent TB infection (LTBI) reactivation and progression of primary pulmonary TB infection in HIV-infected individuals as compared to corresponding rates in those without HIV infection (45). Furthermore, each year 10% of untreated HIV-infected patients with LTBI acquire active pulmonary TB, whereas HIV-uninfected individuals only have a 5-10% lifetime risk of contracting this disease (46). Notably, Th1 and Th17 cells are the main anti-TB effector CD4+ T cells, of which Th1 cells play a protective role by engaging in sustained secretion of IFN-γ, while Th17 cells induce neutrophilic inflammation that damages host tissues. Meanwhile, regulatory T cells engage in negative regulation of T cell responses to prevent excessive inflammation, while Th2 cells mediate humoural immunity and promote an anti-inflammatory response by producing IL-4 and IL-13. Ultimately, a proper balance between T cell types is needed to support the development of TEMs as a source of memory T cells and effector cells that can successfully reduce Mtb load. Such a balance can be obtained *via* BCG vaccination, which generates a large population of TEMs (39).

Regardless, classic TB studies conducted in mice have shown that pulmonary Mtb infection can delay host T cell immune responses (47). Following initial lung infection, APCs migrate from Mtb-infected lung tissue sites to thoracic draining lymph nodes, where they present antigens to naive T cells. These T cells then differentiate into effector T cells that traffic to the lung to eradicate the Mtb infection there. However, Mtb infection can delay the anti-TB immune response by slowing the execution of two steps of the response: 1) transit of APCs from the lung to the lymph node and 2) effector T cell priming and trafficking to the lung.

Mounting evidence has shown that transport of Mtb to mediastinal lymph nodes is initiated at 11–12 days after initial infection (48). However, during the early phase of infection, Mtb delays bacterial transport to lymph nodes by suppressing macrophage apoptosis and Mtb antigen presentation (49, 50). In turn, delayed lung recruitment of Th1 cells leads to impaired T cell priming and decreased chemokine responses within the lung that provide time for Mtb to proliferate. These results suggest that the rate of lung tissue colonisation by IFN-γ-producing CD4+ T cells, not the size of the peripheral CD4+ T cell population, is a crucial feature of protective immunity.

Once Mtb organisms within phagocytic cells are transported from the lungs to the pulmonary lymph nodes, they stimulate proliferation of Mtb-specific Treg cells (51). Consequently, even small numbers of these cells within pulmonary lymph nodes can prevent effector T cell priming from occurring there, which ultimately reduces primed effector T cell accumulation within the lungs (52). Therefore, the abovementioned scenario may explain how a delayed effector T cell immune response can enable early-stage bacterial growth that results in increased bacterial burden in Mtb-infected hosts. Meanwhile, Mtb can affect immune suppression by increasing the number of Tregs to thereby interfere with CD4+ T cell function and TB control in patients with active TB or LTBI. Moreover, Mtb can manipulate host Th cell polarisation that leads to an immune response shift from a Th1 response to a Th2 response (53). Importantly, a Th2 response cannot effectively maintain sustained control over Mtb growth and thus facilitates Mtb survival that perpetuates active TB as a long-term chronic condition. Alternatively, long-term chronic infections and cancer may induce sustained T cell expression of inhibitory cell surface receptors, such as PD-1, TIM-3, LAG-3, and CTLA-4, which can induce T cell exhaustion. T cell exhaustion is a state of reduced effector T cell function that affects both CD4+ and CD8+ T cells, as has been demonstrated by Jayaraman et al. using a murine TB model (54). T cell exhaustion that occurs during chronic Mtb infection is characterized by T cell expression of various inhibitory receptors and gradually decreasing production of IL-2, IFN-γ, and TNF-α (55). Consequently, these dysfunctional T cells lose the ability to clear pathogens and thus are unable to provide effective protection from infection. In addition, exhausted CD4+ T cells release lesser quantities of IFN-γ, a cytokine required for macrophage activation (56), innate immune function, and enhancement of the CD8+ T cell response. In fact, IFN-γ has been shown to boost CD8+ T cell production of IFN-γ and IL-2 that signal immune system detection of Mtb-infected macrophages (57). Finally, T cell dysfunction has been reported to be closely associated with attenuation of BCG vaccine efficacy during the chronic phase of TB infection (58). The mechanisms underlying T cell exhaustion in cancer and TB are similar, and both diseases can cause T cells to lose their ability to function effectively. To overcome T cell exhaustion in cancer, various approaches have been proposed, including checkpoint inhibitors and cytokine-based therapies (59, 60). Checkpoint inhibitors are a type of immunotherapy that block specific immune checkpoint pathways, such as PD-1 and CTLA-4 (61). By blocking these pathways, checkpoint inhibitors restore T cell function and allow them to attack cancer cells. Cytokine-based therapies, such as interleukin-2 (IL-2), IL-7 and IL-15, stimulate the growth and activation of T cells and help restore their function. To treat T cell exhaustion in TB, various approaches are also being developed, including immunomodulatory drugs, such as interferon-gamma (IFN-γ), and immune checkpoint inhibitors (62–64). Additionally, combination therapies, such as the use of antibiotics and immunotherapy, are being explored as a way to restore T cell function and improve treatment outcomes (65, 66).

## Novel TB vaccine challenges and opportunities

In order to induce long-term immunological memory to achieve protection against Mtb infection, efforts are under way to develop new TB-preventive vaccines, such as whole-cell vaccines and subunit vaccines. Despite the fact that an estimated 16 new TB vaccine candidates have entered clinical trials (**Table 2**), these vaccines are unlikely to serve as effective replacements for currently administered BCG vaccines. In this section, we summarize the advantages of new vaccine candidates, as well as their associated challenges and progress toward developing improved vaccines. Two types of viable whole-cell vaccines are currently under development, recombinant BCG (rBCG) vaccines and live attenuated Mtb vaccines. Currently, both MTBVAC (live attenuated Mtb vaccine) and VPM1002 (rBCG vaccine) are enrolled in clinical trials and have been shown to elicit superior anti-Mtb immune responses and protection from infection than are elicited by BCG vaccination. In fact, infants vaccinated with MTBVAC exhibited antigen-specific CD4+ and Th1 cell-mediated inflammatory responses that peaked about 2.5 months post-vaccination, thus demonstrating that a durable memory response was induced that persisted until 12 months post-vaccination (67). In addition, VPM1002 generated higher proportions and numbers of TCM cells to thereby confer stronger protection against Mtb infection than was elicited by the BCG vaccine (37).

**Table 2:** TB vaccine candidates in clinical trials.

Vaccine Candidate	Clinical trial phase	Vaccine type	Current design applications	References
MVA85A	Phase 2b	Viral-vectored vaccines	Prophylactic vaccines	(13)
Ad5Ag85A	Phase 1	Viral-vectored vaccines	Prophylactic vaccines	(14)
VPM1002	Phase 3	Recombinant BCG vaccines	Prophylactic vaccines	(15)
MTBVAC	Phase 2a	Live,attenuated Mtb vaccines	Prophylactic vaccines	(16)
AEC/BC02	Phase 1	Adjuvanted protein subunit vaccines	Prophylactic and therapeutic vaccine	(17)
RUTI	Phase 2a	Killed vaccae vaccines	Therapeutic vaccines	(18)
TB/FLU-04L	Phase 2a	Viral-vectored vaccines	prophylactic vaccines	(19)
DAR-901	Phase 2b	Killed vaccae vaccines	Prophylactic and therapeutic vaccine	(20)
M72/AS01E	Phase 2b	Adjuvanted protein subunit vaccines	Prophylactic and therapeutic vaccine	(21)
H56:IC31	Phase 2b	Adjuvanted protein subunit vaccines	Prophylactic vaccines	(22)
ID93+GLA-SE	Phase 2a	Adjuvanted protein subunit vaccines	Prophylactic vaccines	(23)
MIP	Phase 3	Killed vaccae vaccines	Therapeutic vaccines	(19)
Vaccae™	Phase 3	Killed vaccae vaccines	Therapeutic vaccines	(19)
GamTBvac	Phase 1	Adjuvanted protein subunit vaccines	Prophylactic vaccines	(24)
H1:CAF01	Phase 1	Adjuvanted protein subunit vaccines	Prophylactic vaccines	(25)
H4:IC31	Phase 2b	Adjuvanted protein subunit vaccines	Prophylactic vaccines	(26)

However, due to their similarities to Mtb, whole-cell and BCG vaccine efficacies can be reduced by host pre-sensitisation by exposure to environmental Mtb. Meanwhile, additional factors can contribute to reduced vaccine efficacy, including delayed Mtb progression, chronic antigen-induced inflammation, Treg induction, and T cell exhaustion. Since live pathogens in whole-cell vaccines can persist in vaccinated recipients for months or even years, the resulting chronic antigen stimulation could promote formation of inflammatory microenvironments that may induce increased proportions of Treg and T cells that are prone to exhaustion. As an alternative, vaccines administered *via* the mucosal route may avoid the delayed Mtb progression conundrum by inducing immune responses at the Mtb point of entry. In fact, results of numerous studies suggest that mucosal BCG vaccines provide more robust protective immunity than vaccines administered *via* standard intradermal immunization (68). Additionally, mucosal administration of MTBVAC to rhesus macaques elicits an immune response with a broader antigenic profile and more robust local immunity than that induced by mucosal BCG vaccines (69).

Although viable whole-cell vaccines are advantageous, since they can elicit broad-spectrum immune responses, they can also trigger undesirable inflammatory responses that increase the risk of adverse side effects. By contrast, subunit vaccines, which provide antigen-specific immunity to only one or a few antigens, must be delivered as either adjuvanted proteins or proteins encoded by viral vectors to achieve enhanced immunity. Notably, a large body of evidence has shown that subunit vaccines can generate robust immunological memory and high proportions of TCM and TRM cells (70, 71). Currently, such vaccines are administered after BCG vaccination to increase BCG-mediated protection and duration of immunity.

Many Mtb antigens have unique expression patterns at different stages of infection (72), which poses a significant challenge for vaccine design, in that they cannot replicate to provide widespread distribution of antigens. This drawback leads to low-level antigen expression that is associated with poor T cell expansion, while induction of high-level prolonged antigen expression by some vaccines has been shown to lead to CD4+ T cell exhaustion in mice and humans (73). Nevertheless, use of effective adjuvanted subunit vaccines can counteract high-level antigen-driven T cell exhaustion by maintaining T cells in a protected, less differentiated state (50). Meanwhile, numerous Mtb antigens are uniquely expressed during different Mtb infection stages that may be incorporated in vaccines to generate broader antigen-specific immunity against Mtb during multiple infection stages. Toward this end, two highly promising recombinant fusion protein subunit vaccines, M72/AS01E and H5, have been developed for use in protecting adults from pulmonary TB infection and are currently under evaluation in clinical trials. Such vaccines require adjuvants such as alum, which is currently the most widely used adjuvant in subunit vaccines. Nonetheless, alum mainly enhances humoural immune responses, not cellular immunity. Thus, novel adjuvants are needed that can enable new TB subunit vaccines to induce cell-mediated Th1-type immune responses. One such adjuvant, IC31, a novel toll-like receptor 9 (TLR9) agonist-based adjuvant, may be suitable for this purpose. When IC31 is administered in combination with H4 antigen, a robust H4-specific IFN-γ response is induced at roughly two weeks post-injection (74). Meanwhile, administration of the liposome adjuvant dimethyldioctadecylammonium/monophosphoryl lipid A/trehalose 6,6′-dibehenate (DMT) with the rRv0572c protein antigen has been shown to enhance BCG immunogenicity to prevent LBTI reactivation (75). Furthermore, a gadoteridol-loaded cationic adjuvant formulation 01(CAF01)-adjuvanted H1 vaccine has been shown to generate significant antigen-specific T-cell responses that were still detectable after three years, thus demonstrating that long-lasting immunological memory against Mtb can be established in vaccine recipients (25).

Alternatively, viral vectors may successfully deliver antigen-encoding genes into host cells to induce T cell-mediated immunity without requiring adjuvants. For example, MVA85A, a modified vaccinia Ankara (MVA) virus that delivers Ag85A antigen, is a promising vectored vaccine candidate that is currently under evaluation in clinical trials. MVA85A promotes TCM and TEM production and dramatically enhances long-term T cell responses in healthy human subjects (76). However, a recent phase IIb trial of MVA85A demonstrated that durable immunological memory elicited by this vaccine was unable to boost BCG-induced protection against TB in infants (76). Additionally, as for adjuvanted protein vaccines, viral vector vaccines may not provide suitable antigen doses since they possess high-level immunogenicity, which tends to drive terminal differentiation of T cells. As a result, virus-vectored vaccines may induce production of lower proportions of TCMs than are generated by adjuvanted protein vaccines.

## Conclusion

Vaccination is one of the most effective interventions for achieving infection prevention and control. The importance of traditional T cell subsets (CD4+ T cells and CD8+ T cells) in the treatment of Mtb infection has been fully recognized, but the efficacy of Mtb vaccine largely depends on the immune memory mediated by Tm cells (77). This review further demonstrates the exact role of memory T cell-mediated immune response in the process of anti-tuberculosis, and summarizes the effective ways to increase the number of T cell subsets, which provides a theoretical basis for the development of an effective new vaccine. Vaccination against Mtb is thought to greatly depend on Tm cell-mediated immunological memory, especially that provided by CD4+ Tm cells. Among various Tm cell subsets, TCMs are thought to play the main role in maintenance of long-term immunoprotection, while TRMs provide superior local immunoprotection and a rapid anti-pathogenic response. However, additional in-depth studies of Tm cells and cell subsets derived from them are needed to guide TB vaccine development, since the large number of promising vaccine candidates that have reached advanced clinical trial stages may not provide adequate long-term protection against Mtb infection. Moreover, although these vaccine candidates provide superior efficacy to BCG when administered to animal TB models and infants, their efficacies when in adults are unknown due to lacking clinical data. Regardless, all vaccines described here, including combined vaccines incorporating viable whole-cell vaccines and subunit vaccines, must be further developed to overcome numerous challenges before they can be administered in clinical settings. In addition, all vaccines must boost proportions of TCM and TRM populations in order to elicit long-term immunity. To achieve these goals, future vaccines must incorporate improved immune adjuvants and delivery platforms based on greater in-depth understanding of host-pathogen interactions. As a final note, vaccine induction of dysfunctional T cell populations and T cell exhaustion should be taken into account while designing vaccines to combat TB.

## Author contributions

YP and JY contributed to the conceptual design. XL and HL contributed to the writing and generation of figures for this manuscript. SL contributed to the editing. All authors contributed to the article and approved the submitted version.
